# Puberty Blockers and Suicidality in Adolescents Suffering from Gender Dysphoria

**DOI:** 10.1007/s10508-020-01743-6

**Published:** 2020-06-03

**Authors:** Michael Biggs

**Affiliations:** Department of Sociology, St Cross College, University of Oxford, 42 Park End Street, Oxford, OX1 1JD UK

According to Turban, King, Carswell, and Keuroghlian (2020), suicidal ideation is lower in transgender adults who as adolescents had been prescribed “puberty blockers”—gonadotropin-releasing hormone analogs (GnRHa). This finding was derived from a large nonrepresentative survey of transgender adults in the U.S., which included 89 respondents who reported taking puberty blockers. Turban et al. (2020) tested six measures of suicidality and three other measures of mental health and substance abuse. With multivariate analysis, only one of these nine measures yielded a statistically significant association: the respondents who reported taking puberty blockers were less likely to have thought about killing themselves than were the respondents who reported wanting blockers but not obtaining them. This finding was widely reported in the media; the lead author published a column on its implications for health policy in the *New York Times* (Turban, 2020).

Unfortunately, the finding came from a low-quality survey which is known to have elicited unreliable answers on puberty blockers. The analysis assumed that puberty blockers were available in the U.S. several years before they actually were. Most seriously, Turban et al. (2020) barely acknowledged the fact that adolescents with severe psychological problems would have been less eligible for drug treatment, which confounds the association between treatment and suicidal ideation. The article therefore provided no evidence to support the recommendation “for this treatment to be made available for transgender adolescents who want it” (Turban et al., 2020, p. 7).^1^

Turban et al. (2020) analyzed data from the United States Transgender Survey of 2015 (James et al., 2016). Respondents were not sampled from any defined population, but were recruited online. This convenience sample excluded those who underwent medical intervention and then subsequently stopped identifying as transgender. Obviously, those who actually committed suicide are omitted. Aside from these general problems with the survey, the key questions on puberty blockers evidently confused many respondents. Puberty blockers are given below the age of 16 years, when adolescents become eligible for cross-sex hormones (Hembree et al., 2009). Yet, 73% of respondents who reported having taken puberty blockers (question 12.9) said they started on them *after* the age of 18 years. As the survey report acknowledged, “the question may have been misinterpreted by some respondents who confused puberty blockers with the hormone therapy given to adults and older adolescents” (James et al., 2016, p. 126). Turban et al. (2020) did not mention this misinterpretation but did follow the report’s mitigation strategy of ignoring those respondents who reported taking puberty blockers after the age of 18. No such adjustment is possible, however, for the question asking whether the respondent had ever wanted puberty blockers (question 12.8), which Turban et al. (2020) used to define the subset of respondents in their analysis. The comparison group therefore included an unknown number of respondents—possibly the majority—who actually wanted cross-sex hormones.

The subsample was confined to respondents who were aged under 18 in 1998, because Turban et al. (2020, p. 3) assumed that they “would have had health care access to GnRHa for pubertal suppression.” GnRHa was first used to treat “juvenile transsexuals” in the Netherlands in the mid-1990s, with the first case study published in 1998 (Cohen-Kettenis & van Goozen, 1998; Gooren & Delemarre-van der Waal, 1996). But it took several years for the U.S. to follow suit. An early advocate was Spack, a pediatric endocrinologist at Boston Children’s Hospital, who remembers “salivating” when he first heard about the Dutch model (Hartocollis, 2015). Although this hospital provided cross-sex hormones from 1998, puberty blockers were not offered until Spack co-founded its Gender Management Service in 2007 (Spack et al., 2012). That was the first specialized gender clinic for children in the U.S. (cf. Zucker, 2015). Only in 2009 did the Endocrine Society recommend puberty suppression for gender identity disorder (as gender dysphoria was then known); Spack helped to write its guidelines (Hembree et al., 2009). “There was an attitudinal shift to be able to say that the Endocrine Society supports this,” he recalled a few years later (Ruttimann, 2013, p. 19). If one had to choose a year from which puberty blockers became generally available in the U.S., it would be 2009. This periodization is supported by complete data on prescriptions of one formulation of GnRHa (histrelin acetate) from 43 children’s hospitals: it was never prescribed for gender identity disorder between 2004 and 2009 and was then prescribed to 92 patients from 2010 to 2016 (Lopez, Solomon, Boulware, & Christison-Lagay, 2018). That also accords with Turban and Keuroghlian’s (2018, p. 451) own statement that transition for young (American) adolescents has been recommended “[d]uring the past 10 years.” Faulty periodization means that Turban et al.’s (2020) subsample included older respondents who, in fact, had no opportunity to obtain these drugs and so cannot be used for comparison.

In discussing their results, Turban et al. (2020, p. 7) briefly admitted the possibility that “those without suicidal ideation had better mental health when seeking care and thus were more likely to be considered eligible for pubertal suppression.” Indeed, the Endocrine Society’s initial guidelines restricted eligibility to adolescents who “[d]o not suffer from psychiatric comorbidity that interferes with the diagnostic work-up or treatment” and “[h]ave adequate psychological and social support during treatment” (Hembree et al., 2009, p. 3138). The revised stipulation is that “[a]ny coexisting psychological, medical, or social problems that could interfere with treatment…have been addressed, such that the adolescent’s situation and functioning are stable enough to start treatment” (Hembree et al., 2017, p. 3878). There is evidence that such guidelines are followed in clinical practice, at least to an extent. Gender Management Service at Boston Children’s Hospital, for example, does not accept patients with severe psychopathology (Ruttimann, 2013). Analysis of 109 adolescents at one clinic demonstrates that patients who reported more psychological and social problems were less likely to receive puberty blockers, controlling for several other factors (Zucker et al., 2011).

Psychological problems are, therefore, a confounding factor that will create a spurious association between suicidality and treatment. The confounding could be resolved only if we could properly measure the respondent’s psychological problems *before* GnRHa was prescribed or withheld.^2^ Without any such measures, a negative association found many years after treatment is compatible with three scenarios: puberty blockers reduced suicidal ideation; puberty blockers had no effect on suicidal ideation; puberty blockers increased suicidal ideation, albeit not enough to counteract the initial negative effect of psychological problems on eligibility. Turban et al. (2020, p. 7) acknowledged that “the study’s cross-sectional design…does not allow for determination of causation.” Such caution was not conveyed in many news reports generated by the study. “Puberty blockers reduce suicidal thoughts in trans people” ran a typical headline (LGBTQ Nation, 2020).

In sum, then, Turban et al. (2020) contributed nothing to our knowledge of the effects of suppressing puberty in adolescents. One study did demonstrate positive psychological effects, based on measures taken from between 41 and 57 individuals, with no control group (de Vries, Steensma, Doreleijers, & Cohen-Kettenis, 2011). A second study cited by Turban et al. (2020) actually showed no statistical difference in improvement in psychological functioning between the group prescribed puberty blockers and the group given therapy (Biggs, 2019; Costa et al., 2015). “Longitudinal clinical trials are needed to better understand the efficacy of pubertal suppression,” as Turban et al. (2020, p. 7) observed. It is remarkable that such a call is necessary nearly a quarter of a century after this treatment was first proposed.

## Footnotes

1 The journal that published the target article, *Pediatrics*, rejected an earlier version of this Letter as an online comment without providing a reason.

2 Parental support and social class are additional confounding factors, though the analysis did control for these—albeit poorly, as the variables pertain to the respondent’s current situation and not their situation before treatment.
