# Correction to: Puberty Blockers and Suicidality in Adolescents Suffering from Gender Dysphoria

**DOI:** 10.1007/s10508-021-02056-y

**Published:** 2021-06-01

**Authors:** Michael Biggs

**Affiliations:** Department of Sociology, St Cross College, University of Oxford, 42 Park End Street, Oxford, OX1 1JD UK

## Correction to: Archives of Sexual Behavior (2020) 49:2227–2229 10.1007/s10508-020-01743-6

The Letter to the Editor “Puberty Blockers and Suicidality in Adolescents Suffering from Gender Dysphoria,” written by Michael Biggs, was originally published electronically on the publisher’s internet portal on 3 June 2020 without open access. With the author’s decision to opt for Open Choice, the copyright of the letter changed on 17 May 2021 to © The Author(s) 2021 and the Letter is forthwith distributed under a Creative Commons Attribution 4.0 International License, which permits use, sharing, adaptation, distribution, and reproduction in any medium or format, as long as you give appropriate credit to the original author and the source, provide a link to the Creative Commons license, and indicate if changes were made. The images or other third party material in this article are included in the article's Creative Commons license, unless indicated otherwise in a credit line to the material. If material is not included in the article's Creative Commons license and your intended use is not permitted by statutory regulation or exceeds the permitted use, you will need to obtain permission directly from the copyright holder. To view a copy of this license, visit http://creativecommons.org/licenses/by/4.0/.

